# Neuromoral Diversity: Individual, Gender, and Cultural Differences in the Ethical Brain

**DOI:** 10.3389/fnhum.2017.00501

**Published:** 2017-10-31

**Authors:** Geoffrey S. Holtzman

**Affiliations:** ^1^Center for the Study of Ethics in the Professions, Illinois Institute of Technology, Chicago, IL, United States; ^2^Center for Translational Bioethics and Healthcare Policy, Geisinger Health System, Danville, PA, United States

**Keywords:** cultural differences, gender differences, individual differences, moral psychlogy, neuroethics, philosophy, RTPJ

The search for the neural correlates of moral judgment has been rapidly expanding in recent years. Perhaps the best-known search technique is neuroimaging, but significant contributions have also been made by fields including neuroendocrinology, neuropharmacology, neuropathology, and neurogenetics. In addition to the value this multidisciplinary search holds for social, cognitive, and affective neuroscience, it has far-reaching implications for philosophy (Greene, 2007), ethics (Tabery, 2009), and law (Saks et al., 2014). While this search has yielded great advances in our scientific understanding of moral judgment over the past decade and a half, one important fact has been underappreciated by philosophers, ethicists, legal scholars, and even many neuroscientists. This is the fact that the neural correlates of moral judgment may be just as diverse as the people making those judgments.

It may not be surprising that people's moral judgments track sociocultural background (Sarkissian et al., 2010), individual differences (Holtzman, 2013), and other factors (Schwitzgebel, 2010). But much more interesting is the fact that the neurobiological resources recruited in the service of moral judgments appear to vary along those same lines. Currently, much (though certainly not all) research in the neuroscience of moral judgment seeks to understand “networks of the so-called ‘moral brain’” (Avram et al., 2014). “The moral brain” typically refers to a theoretic construct that is partly premised on the idea that by studying how some brains formulate moral judgments, we can come to understand how brains in general do.

While a unified conception of “the moral brain” may be valuable to some extent, there is also an extent to which a truly comprehensive, scientific, and morally and philosophically justifiable conception of neuromoral processing must always keep in mind the nature and implications of variability between persons—and between their moral neurophysiologies. This is especially important in light of the fact that the overgeneralization of claims about the nature of moral judgment can raise not only scientific concerns, but also ethical and philosophical worries. I review evidence suggesting that there exists a wide variety of ways in which moral judgments may be realizable in human neural architecture. From this point of departure, I address just a few of the many implications that such neuromoral diversity—and the failure to fully appreciate neuromoral diversity—may have in philosophical, ethical, and legal contexts.

## Individual differences in the neuroscience of moral judgment

The involvement of some brain circuitry in moral processing varies depending on individual difference factors. Consider the right temporoparietal junction (rTPJ), a region whose involvement in moral judgment (Young et al., 2007, 2010; Young and Saxe, 2009) appears to be related to its role in understanding others' mental states (Saxe and Kanwisher, 2003; Saxe and Wexler, 2005). Yoder and Decety (2014) recently found that individual differences in justice sensitivity predicted differences in activation of the rTPJ among participants viewing video of people interacting in moral contexts. In addition to the differences in *activation* they discovered, they also found that individual differences in justice sensitivity predicted differences in crucial (Miller et al., 2010) *coupling* between the rTPJ and other brain regions.

This is not necessarily to say that individual differences in justice sensitivity cause differences in rTPJ activity; the arrow of causation may point in the other direction, or there may be some third variable at work. My point in highlighting these findings is just that any sweeping description of the role of the rTPJ in moral judgment may entail oversimplifying the relationship between neural structures like the rTPJ on the one hand, and moral judgment on the other.

Findings like these also suggest that a shift toward research that emphasizes individual differences could connect with and enhance other ongoing paradigm shifts in the neuroscience of moral judgment, and in neuroscience more generally. In particular, such findings seem to bolster longstanding arguments pertaining to the need to shift the focus of research away from individual brain regions, and toward neural networks.

Yoder and Decety's (2014) findings may also have important implications for questions in philosophical ethics that, until recently, have been approached almost entirely from the armchair. For instance, Nagel (1976) introduced important questions about the role of luck in moral standing. “Constitutive moral luck,” as philosophers call it, concerns the external forces that give individuals their unique “dispositions of morality” (Williams, 1981). Seen through the lens of constitutive moral luck, neuromoral diversity can contribute new, important perspectives to longstanding debates about the extent to which people can or should be held morally responsible for actions that, in part, reflect unchosen differences in their neurobiological makeup and the factors that may have shaped it.

## Gender differences in the neuroscience of moral judgment

Regions within the ventral prefrontal cortex, including the ventromedial prefrontal cortex and the frontopolar cortex (Bryant et al., 2016; Garrigan et al., 2016), have been widely associated with certain kinds of moral judgment. Recently, Fumagalli et al. (2010a,b) found positive electrical stimulation of the ventral prefrontal cortex to increase women's judgments of moral permissibility for personal harms that increased overall welfare. However, they found no such effect on men's judgments. Likewise, negative stimulation of the area decreased permissibility ratings provided by women, but did not affect ratings provided by men.

Similarly, Scheele et al. (2014) reported that intranasal administration of oxytocin increased moral approbation among men, but did not affect the moral judgments reported by women (although cf. Walum et al., 2016). And Harenski et al. (2008) found that in female subjects, severity of moral judgment was modulated by posterior cingulate gyrus and insula activity, whereas males showed a stronger relationship between severity of moral judgment and inferior parietal activity. More recently, Harenski et al. (2014) found that male psychopaths exhibited normal rTPJ activity when viewing pictures of moral transgressions, but that rTPJ activity among over 200 female psychopaths was reduced during this task.

Collectively, these findings suggest that our gendered social institutions and structures (Friedman, 1987), and the identities they impose on individuals, may have profound implications for the ways different people process moralistic situations at the biological level. Non-essential physiological differences between persons (namely, physiological differences developed in response to social-environmental factors) may therefore have a major impact on moralistic behaviors and responses. We need to consider this deeply in deciding to what extent we hold persons responsible for these behaviors and responses.

Critically, these studies also reveal how research pertaining to gender differences in the neuroscience of moral judgment may be at especially high risk for being misinterpreted or misrepresented in ways that exacerbate previously-recognized issues of “neurosexism” (Fine, 2010; Bluhm, 2013). For instance, Harenski et al.'s contributions to our understanding of the biological bases of moral judgment are interesting and important, but one should be hesitant to repeat their assertion that female participants showed “increased activity in regions associated with care-based processing” (Harenski et al., 2008). Their use of the term “care-based processing” was innocently borrowed from research that was not concerned with gender (Robertson et al., 2007), but the term has problematically gendered connotations, and is nowhere else used in neuroscience literature.

## Cultural differences in the neuroscience of moral judgment

A great deal of research on the neuroscience of moral judgment, beginning with Greene et al. (2001), has studied differences in the neural resources recruited in the judgment of “personal” moral transgressions involving gratuitous physical harm to specific persons, in contrast to “impersonal” transgressions that do not involve this. In cross-cultural research, Han et al. (2014) replicated Greene et al.'s earlier (2001) finding that the medial frontal gyrus is more strongly activated by “personal” than “impersonal” moral vignettes among American participants, but found this pattern to be absent among Korean participants. The same cross-cultural patterns of difference were found in the anterior cingulate cortex, for which Han et al. (2014) again replicated Greene et al.'s (2001) findings among Americans, but failed to replicate those findings among Korean participants. In still other areas, Han et al. (2014) confirmed Greene et al.'s (2001, 2004) null findings among American participants, but found significant effects among Korean participants. Other research has found similar differences between Chinese and American participants in not only the topology of neuromoral processing, but also its time-course (Wang et al., 2014). Both long- and short-term acculturative processes likely contribute to these differences.

Differences in neuromoral processing that track religion (Christensen et al., 2014) and other community values (Telzer et al., 2010) may sometimes emerge during the (relatively short) period of individual lifetimes. In contrast, other variability in moralistic behavior may have an evolutionary basis. Gelfand et al. (2011) have amassed evidence suggesting that relatively strict, *tight* (Pelto, 1968) adherence to norms may confer a survival advantage among cultures facing significant ecological threats. Considered alongside evidence that serotonin plays an important role in maintaining normative moral behavior and judgment (Crockett et al., 2008) by increasing harm aversion (Crockett et al., 2010; cf. Crockett, 2014), this suggests that allelic differences in the serotonin transporter gene (5-HTTLPR) might partly mediate the relationship between cultural lineage and tightness of moral judgment. Indeed, research exploring this hypothesis is already being conducted (e.g., Mrazek et al., 2013).

Cross-cultural differences, such as those identified by Han et al. (2014) and Wang et al. (2014), may have important implications for both the neuroscience of moral judgment and for philosophical ethics. From the perspective of neuroscience, it may not be feasible to develop a unified picture of the neural correlates of moral judgment if ecological pressures have led the relevant neural structures to develop in a variety of different ways. Furthermore, findings about “the moral brain” may in many cases only be findings about specific cross-sections of the Western, well-educated, wealthy brain (Henrich et al., 2010). From a more philosophical perspective, we can see that it may be both ethnocentric and factually inaccurate to characterize in strong moral terms culturally engrained tendencies that may reflect evolutionary pressures rather than individual scrupulosity or laxness in moral character. If so, this exacerbates already-vexed philosophical and practical questions about the extent to which we can and should hold people responsible for acting on those tendencies.

The potential for neurogenetic heritage to influence moral behavior and judgment also raises important questions at the intersection of ethics and law. Courtrooms around the world have begun considering the admissibility of neurogenetic evidence that, some defense teams have claimed, mitigates legal culpability for violent crimes (Forzano et al., 2010). Evaluating the legitimacy of such claims is inherently complicated, and involves many kinds of research, including research in neurolaw and neurobiology. But it becomes even more difficult once one recognizes that neurogenetic variance in prosocial and antisocial behavior and judgment, which *prima facie* may not appear to be ethically fraught, may be intimately related to cultural differences closely associated with moral practice. Understanding and accommodating such cultural differences in scientific but non-essentialist ways is a difficult matter that raises a number of ethical concerns related to ethnocentrism. This is yet one more matter that scholars have yet to devote significant attention to, but which we will very likely have to consider more fully in the near future.

## Discussion

All of the findings discussed here add further complexity to a picture of neuromoral processing that other research has already suggested is extraordinarily complex. More researchers should follow the trail blazed by the authors mentioned in this essay, and consider what the neuroscience of moral judgment might look like if we were to free ourselves from the idea that we should be developing a unified picture of “the moral brain.” Such a shift will also enable greater integration of important research into the effects of situational factors (Avram et al., 2014) and of factors that distinguish types of moral violations (Parkinson et al., 2011; Abend, 2013) on neuromoral processing. We should pursue the thought that moral judgment is a multifaceted and multiply realizable affair, and that the neural circuitry underwriting such judgment is also multifaceted, multiply realizable, and variable across cultures, individuals, and situations. This is the only ethical way forward, and it is also the path most likely to bear scientific fruit.

## Author contributions

The author confirms that he is the sole contributor to this work, and approved it for publication.

### Conflict of interest statement

The author declares that the research was conducted in the absence of any commercial or financial relationships that could be construed as a potential conflict of interest.
